# Physicochemical, Nutritional, and Structural Characterization of a Novel Meat-Based Hummus

**DOI:** 10.3390/foods14142507

**Published:** 2025-07-17

**Authors:** Meena Goswami, Rishav Kumar, Xin M. Teng, Ravi Jadeja, Darren Scott, Morgan Pfeiffer, Gretchen G. Mafi, Vikas Pathak, Ranjith Ramanathan

**Affiliations:** 1 Department of Animal and Food Sciences, Oklahoma State University, Stillwater, OK 74078, USA; rishav.kumar@okstate.edu (R.K.); ravi.jadeja@okstate.edu (R.J.); morgan.pfeiffer@okstate.edu (M.P.); gretchen.mafi@okstate.edu (G.G.M.); 2 Department of Livestock Products Technology, College of Veterinary Science & Animal Husbandry, DUVASU, Mathura 281001, India; pathakvet@gmail.com; 3 Robert Kerr Food and Agricultural Products Center, Oklahoma State University, Stillwater, OK 74078, USA; xteng@okstate.edu (X.M.T.); darren.scott@okstate.edu (D.S.)

**Keywords:** meat-based hummus, minced mutton, functional food, sodium acid sulfate, food safety

## Abstract

The objective was to characterize physicochemical, nutritional, and structural properties of a novel meat-based hummus. This product was created by substituting 50% of chickpea paste with mutton. The meat-based hummus contained 0.4% sodium acid sulfate as an antimicrobial agent. The pH values of traditional hummus were greater than those of the meat-based hummus. There was no significant difference in day 0 total plate count between plant- and meat-based hummus; however, the total plate count on day 7 was significantly (*p* < 0.05) lower in the meat-based hummus than plant-based hummus due to antimicrobial addition. Instrumental color analysis showed greater lightness (L* values) and yellowness values for traditional hummus compared to the meat-based hummus. The meat-based hummus had 66% greater protein than traditional hummus. Scanning electron microscopy revealed a porous, gel-like structure in plant-based hummus, while meat-based hummus showed a dense, fibrous network. The flavor, creaminess, grain properties, and mouth coating scores of meat-based hummus were greater than those of traditional chickpea hummus. The study indicated that meat-based hummus can be developed by incorporating 50% cooked minced mutton. Creating innovative meat-based products like meat hummus offers the benefits of both plant-based and animal-based diets, making it a good option for flexitarians.

## 1. Introduction

Hummus is a traditional Middle Eastern delicacy that has gained worldwide popularity for its rich flavor and nutritional profile [1]. It is typically made by blending cooked chickpeas with tahini, olive oil, lemon juice, garlic, and salt and functions as a nutritious, plant-based food that provides a balanced macronutrient profile. Chickpeas provide complex carbohydrates and plant-based proteins, while tahini and olive oil contribute essential monounsaturated and polyunsaturated fatty acids, which are known to support cardiovascular health and modulate inflammation [2]. Moreover, hummus is a good source of dietary fiber, aiding digestion and glycemic control, and contains vital micronutrients such as iron, magnesium, folate, and potassium, each playing key physiological roles.

Hummus is used as a dip, a spread on toast or sandwiches, or even as a base for salad dressings and sauces. However, this ready-to-eat hummus is high in sodium, sometimes providing over 400–600 mg per serving, which significantly contributes to daily sodium intake and may lead to elevated blood pressure and increased cardiovascular risk [3]. Another concern is allergenicity. For example, allergens such as soy, wheat gluten, or pea protein may provoke allergic reactions ranging from mild gastrointestinal discomfort to severe anaphylaxis in sensitive individuals [4]. Additionally, commercially available ready-to-eat hummus is often fortified with synthetic nutrients such as iron, vitamin B_12_, and zinc. While this may seem beneficial, the bioavailability of such nutrients is often lower than that from animal sources, and imbalanced intake may lead to nutritional excess or poor absorption over time [5]. Furthermore, chickpeas, the main ingredient in hummus, are high in fermentable carbohydrates (FODMAPs), which can trigger bloating, gas, and discomfort in individuals with irritable bowel syndrome. These symptoms may be exacerbated when combined with other high-fiber or soy-based ingredients [6]. To the best of our knowledge, there have been limited attempts to create a meat-based hummus.

Integrating lean minced mutton into hummus presents a novel opportunity to address the micronutrient deficits inherent in plant-based formulations. Red meat, especially mutton, is a source of bioavailable heme iron and vitamin B_12_. These nutrients are essential for populations with increased physiological demands, such as pregnant women, children, athletes, and the elderly [7]. This fusion of legumes and meat may offer a nutritionally complementary matrix, blending fiber and phytochemicals with complete amino acids and absorbable minerals. Hummus is highly susceptible to foodborne pathogens, such as *Salmonella*, *E. coli*, and *Listeria* [8,9,10]. From 2002 to 2017, hummus was linked to 17 foodborne illness outbreaks in the United States, which collectively resulted in 1105 reported cases, 64 individuals requiring hospitalization, and four fatalities [11]. Incorporating meat into chickpea hummus may render it highly perishable due to its higher moisture content and nutrient-rich environment, making it vulnerable to microbial proliferation. Sodium acid sulfate (SAS), also known as sodium bisulfate, has been Generally Recognized as Safe (GRAS) by the U.S. Food and Drug Administration since 1998. More recently, the U.S. Environmental Protection Agency (EPA) listed SAS as a “Safer Choice” antimicrobial and processing aid/additive due to its effectiveness and safety profile [12]. Studies have demonstrated its antimicrobial efficacy; for instance, a 15 s dip in 1.0–3.0% SAS solution reduced *Salmonella enteritidis* on whole chicken drumsticks by 0.9–1.6 log_10_ CFU/g [13], while a 90 s spray application of 10.0% SAS lowered *Salmonella typhimurium* levels on chicken carcasses by 2.3–2.6 log_10_ CFU/bird [14]. Sodium acid sulfate can act as both an acidulant and an antimicrobial agent, reducing pH and disrupting microbial membranes, thereby suppressing the growth of spoilage and pathogenic organisms [9,15]. Hummus has a relatively high moisture level and a neutral pH. Thus, incorporating SAS can be particularly effective in reducing microbial growth and minimizing the risk of foodborne illness [9]. While the concept of combining meat with traditional hummus may appear incremental, the development of a hybrid hummus formulation incorporating cooked minced mutton and sodium acid sulfate (SAS) offers a novel approach to enhancing both the nutritional and microbiological safety profiles of a traditional vegetarian product. Therefore, the present study aimed to develop and evaluate a novel formulation of meat-based hummus enriched with minced mutton, incorporating sodium acid sulfate as a natural preservative.

## 2. Materials and Methods

### 2.1. Raw Material Processing

Fresh lamb legs from market-age animals were sourced from the Robert M. Kerr Food and Agricultural Products Center, a USDA-inspected facility at Oklahoma State University. The approximate lean level was 85%. The legs from three lambs were deboned and cut into small cubes and pooled. Vacuum-packaged meat samples were stored at −19 °C until further processing. Food-grade sodium acid sulfate (NaHSO_4_) was obtained from Jones-Hamilton Co., based in Walbridge, OH, USA. Chickpeas, sesame seeds, olive oil, salt, garlic, and lemons were purchased from a retail store in Stillwater, Oklahoma. The reagents and chemicals used for analysis were supplied by SRL, Fisher Scientific, CDH, HiMedia, and Sigma-Aldrich (St. Louis, MO, USA).

### 2.2. Hummus Preparation

Chickpea seeds were soaked in water overnight to facilitate softening, followed by cooking in a pressure cooker for 25–30 min to ensure proper texture. Frozen lamb meat was thawed overnight at 4 °C, cut into small pieces, and cooked using a pressure cooker for 20 min under steam without pressure. Tahini paste was prepared by lightly toasting sesame seeds and blending them with olive oil in a kitchen grinder. Pre-weighed quantities of all ingredients, i.e., cooked chickpeas, pressure-cooked lamb, tahini, salt, olive oil, freshly minced garlic, lemon juice, and water, were blended into a uniform, smooth paste. The granules of antimicrobial SAS were pulverized and incorporated into the meat-based hummus, followed by thorough mixing to ensure uniform distribution. The resulting hummus was packaged in pre-sterilized airtight polyethylene terephthalate (PET) containers (GUSTO Small Plastic Cups with Lids Portion Cups & Dessert Cups, 2 oz, 2.8 × 6.0 × 2.8 cm^3^; Comfy package, NY) and stored at refrigeration temperatures (4 ± 1 °C). The various treatments in this study included traditional hummus made with boiled chickpea paste (0% SAS) and meat-based hummus, which replaced 50% of the boiled chickpea paste with 50% minced lamb, along with the addition of 0.4% sodium acid sulfate as a preservative. The overall goal was to prepare a meat-based hummus. Traditional hummus was also used to assess physicochemical and structural properties. Traditional hummus does not contain sodium acid sulfate; therefore, the two products were not compared. The formulation of traditional and meat-based hummus is shown in Table 1.

### 2.3. Product Evaluation

#### 2.3.1. pH

The pH of the freshly prepared hummus was measured using a Hach HQd Portable pH Meter (Loveland, CO, USA). The prepared hummus samples were poured into a beaker, and the pH was measured by immersing the pH probe into the hummus. The pH electrode was washed thoroughly before use. The pH meter was calibrated with pH 4 and 7 buffers before use. Triplicate readings of each sample were taken.

#### 2.3.2. Total Plate Count

Microbiological quality was evaluated by total plate count (TPC) on day 0 and day 7 [9]. For day 0 analysis, samples were taken from freshly prepared hummus, which was then filled into pre-sterilized airtight PET containers. These containers were sealed and stored at 4 °C for day 7. A 10 g portion of each sample was aseptically transferred into 90 mL of pre-sterilized 0.9% saline solution in Whirl-Pak bags and homogenized using a stomacher to obtain a 10^−1^ dilution. Various serial dilutions, from undiluted to 5, were prepared by transferring 1 mL of the homogenized solution into 9 mL of sterile 0.9% saline solution. One mL aliquots from each dilution were plated in duplicate on sterile Petri dishes containing solidified Nutrient Agar (autoclaved at 15 psi for 20 min), spread using a sterile L-shaped spreader, and incubated at 37 °C for 24–36 h. Colonies (30–300) were counted using a colony counter, and TPC was expressed as log cfu/g of the sample.

#### 2.3.3. Instrumental Color Analysis

Instrumental color was measured quantitatively using a portable, reflected-color measurement spectrophotometer. The L* (lightness indicator), a* (redness indicator), and b* (yellowness indicator) values [16] were recorded using a HunterLab MiniScan^®^ EZ 4500L [17]. The HunterLab MiniScan spectrophotometer was standardized before each use with white and black tiles. A transparent polyvinyl chloride film was placed over the aperture of the spectrophotometer to protect the hummus paste from coming in contact with the spectrophotometer sensor. The readings were taken by placing the device in contact with the surface of the hummus after opening the lid of the PET container. Triplicate readings were taken for each sample.

#### 2.3.4. Nutritional Fact Panel

The nutritional content and fact panel of the products were developed using Genesis R&D Food Formulation and Labeling software (ESHA Research, Salem, OR, USA; https://esha.com/products/genesis-rd-food-labeling-software/, accessed on 2 February 2025). This nutritional label software is used in the industry and is also compliant with United States Food and Drug Administration regulations. Previous studies have also utilized this software to determine the nutritional content of various food products [18,19,20].

#### 2.3.5. Scanning Electron Microscopy

Microscopic structural characterization of the meat- and plant-based hummus was done at the Advanced Technology Research Center, Oklahoma State University, Stillwater, using an Aspex Explorer Automated Scanning Electron Microscope (SEM) with energy-dispersive spectroscopy (EDS) technology. Samples from day 0 were used for scanning electron microscopic studies. The samples were freeze-dried, mounted, and sputter-coated with Au (approximately 200 nm) in an SPI diode sputtering system metallizer. The images were taken at magnifications ranging from 60× to 1500× (corresponding to 1 mm to 50 µm).

#### 2.3.6. Sensory Evaluation

Sensory evaluation protocols were approved by the Institutional Review Board (IRB) at Oklahoma State University. Sensory evaluation was conducted using a nine-point hedonic scale, where 9 indicated “extremely desirable” and 1 indicated “extremely poor” [21]. Desirability testing was conducted by seven consumer panel members, who assessed the desirability of the product based on key quality attributes, including color and appearance, flavor, creaminess, grain properties, and mouthfeel. Instructions were given verbally about each sensory trait. Freshly prepared hummus samples were served at approximately 40 °C in a sensory evaluation room during a late-morning session, around 11:00 a.m. The panelists were given plain lukewarm water to rinse their mouths between samples to minimize carryover effects. The sensory evaluation was replicated three times.

### 2.4. Statistical Analysis

A completely randomized design was employed in this study. Each experiment was conducted three times, with duplicate samples taken for each parameter. The total plate count data included storage time. The data obtained from the study on various parameters were statistically analyzed using the SPSS-20.0 software package. The data were subjected to a t-test to compare the F-value and level of significance between samples for total plate count. The significance level was set at *p* < 0.05.

## 3. Results and Discussion

Response surface methodology was used to optimize the level of meat used in the meat-based hummus [22]. For example, several studies were conducted to optimize the level of meat that can replace 25–75% boiled chickpea paste with cooked minced lamb. Similarly, various trials were conducted to investigate the addition of sodium acid sulfate (SAS) as a preservative to extend the shelf life of meat-based hummus [22]. Therefore, optimized levels of meat and sodium acid sulfate were used in this research. A meat-based hummus was prepared by substituting 50% of the boiled chickpea paste with minced mutton, which was cooked for 20 min under steam without pressure, and by adding 0.4% sodium acid sulfate as a preservative.

### 3.1. Physicochemical Properties

**Figure 1:**
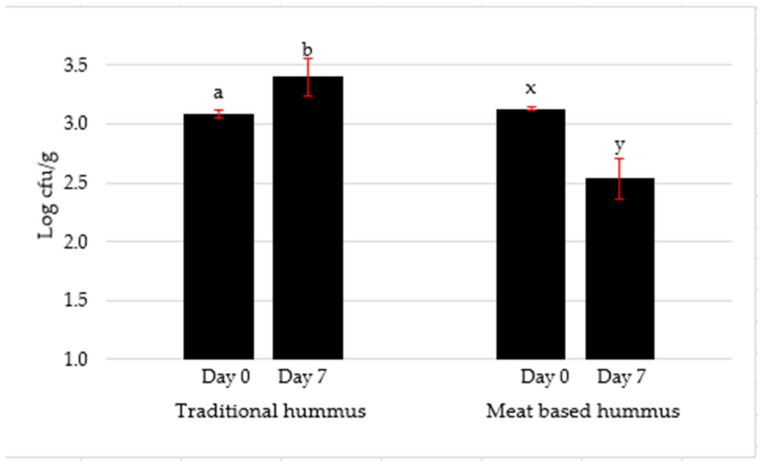
Effects of storage time (days) on total plate count of traditional and meat-based hummus. Means with standard deviation bars are indicated. Comparisons are within the hummus type for days of storage. Different superscript ^a–b^ in traditional or ^x–y^ in meat-based hummus differ significantly (*p* < 0.05).

The physicochemical properties of traditional and meat-based hummus are presented in Table 2. The pH of traditional hummus was comparatively higher than that of meat-based hummus, which might be due to the acidic influence of sodium acid sulfate. Instrumental color analysis showed higher lightness (L*) and yellowness (b*) values in chickpea than meat-based hummus due to natural pigmentation from chickpea starch and leguminous fibers, while the darker hue of meat-based hummus might be attributed to the myoglobin content of lamb and pH-induced pigment denaturation while cooking [23]. There was no difference in redness (a*) values between traditional and meat-based hummus. A previous study also evaluated the color characteristics of traditional hummus [8]. There was no significant difference between traditional and meat-based hummus on day 0 TPC (Figure 1). However, TPC on day 7 was significantly (*p* < 0.05) lower in meat-based hummus, which confirmed the action of sodium acid sulfate on microorganisms with the storage period. SAS enhanced microbial stability by lowering the pH below the optimal growth range for spoilage bacteria [9,24]. Traditional hummus showed an increase from 3.09 to 3.40 log CFU/g, while meat-based hummus showed a decline from 3.13 to 2.54 log CFU/g, suggesting antimicrobial effectiveness in the meat-based hummus treated with 0.4% sodium acid sulfate due to the acidified environment [25]. Future research on the effects of storage on physicochemical properties will help understand how pH and color change with storage time.

### 3.2. Nutritional Fact Panel

The nutritional fact panel of traditional and meat-based hummus is presented in Table 3 and Table 4. A comparative nutritional evaluation of traditional hummus and meat-based hummus revealed marked differences in macronutrient composition, total calories, and functional health ingredients. The meat-based formulation showed a 12.5% increase in total calories (90 kcal vs. 80 kcal per 32 g), a 66.6% increase in protein (5 g vs. 3 g), and a 20% increase in total fat (6 g vs. 5 g) in meat-based hummus than traditional hummus. Although the levels were minimal, saturated fat (1 g vs. 0.5 g) and cholesterol (10 mg vs. 0 mg) content were greater in meat-based hummus compared to traditional hummus. These enhancements are attributed to the inclusion of lamb, which is naturally higher in saturated lipids and complete proteins rich in essential amino acids. Animal protein offers higher biological value and better digestibility compared to plant-based proteins [26]. Additionally, although both products provided 1 mg of iron per serving, meat-based hummus is likely superior in iron bioavailability due to the presence of heme iron, which has a significantly higher absorption efficiency compared to non-heme iron found in legumes [27]. Conversely, traditional hummus provides higher total carbohydrates (7 g vs. 4 g), dietary fiber (2 g vs. 1 g), and calcium (17 mg vs. 14 mg), as chickpeas are an excellent source of complex carbohydrates, resistant starch, and prebiotic fiber [28]. The modest increase in sodium (150 mg vs. 130 mg) in meat-based hummus may be attributed to both the intrinsic sodium content of meat and the addition of sodium acid sulfate, which serves as both an acidulant and a preservative. While meat-based hummus improves protein and iron bioavailability, it has a moderate increase in saturated fat and cholesterol levels [29,30]. Therefore, meat-based hummus can be a nutritious choice for individuals seeking higher-quality protein and greater iron bioavailability, such as athletes, growing children, or the elderly. When incorporated into a balanced diet, meat-based hummus can support muscle maintenance and overall nutritional adequacy, especially in populations with increased protein needs or concerns about iron deficiency.

Table 4 summarizes the nutritional information for 100 calories of both traditional and meat-based hummus. A similar trend was observed when the nutritional profile of traditional and meat-based hummus was compared, with a serving size of 32 g. There was a 48% increase in protein content among those who consume food based on calorie requirements. Additionally, meat-based hummus combines the benefits of both plant-based and animal-based diets, making it a good choice for those following a flexitarian diet.

### 3.3. Scanning Electron Microscopy

**Figure 2:**
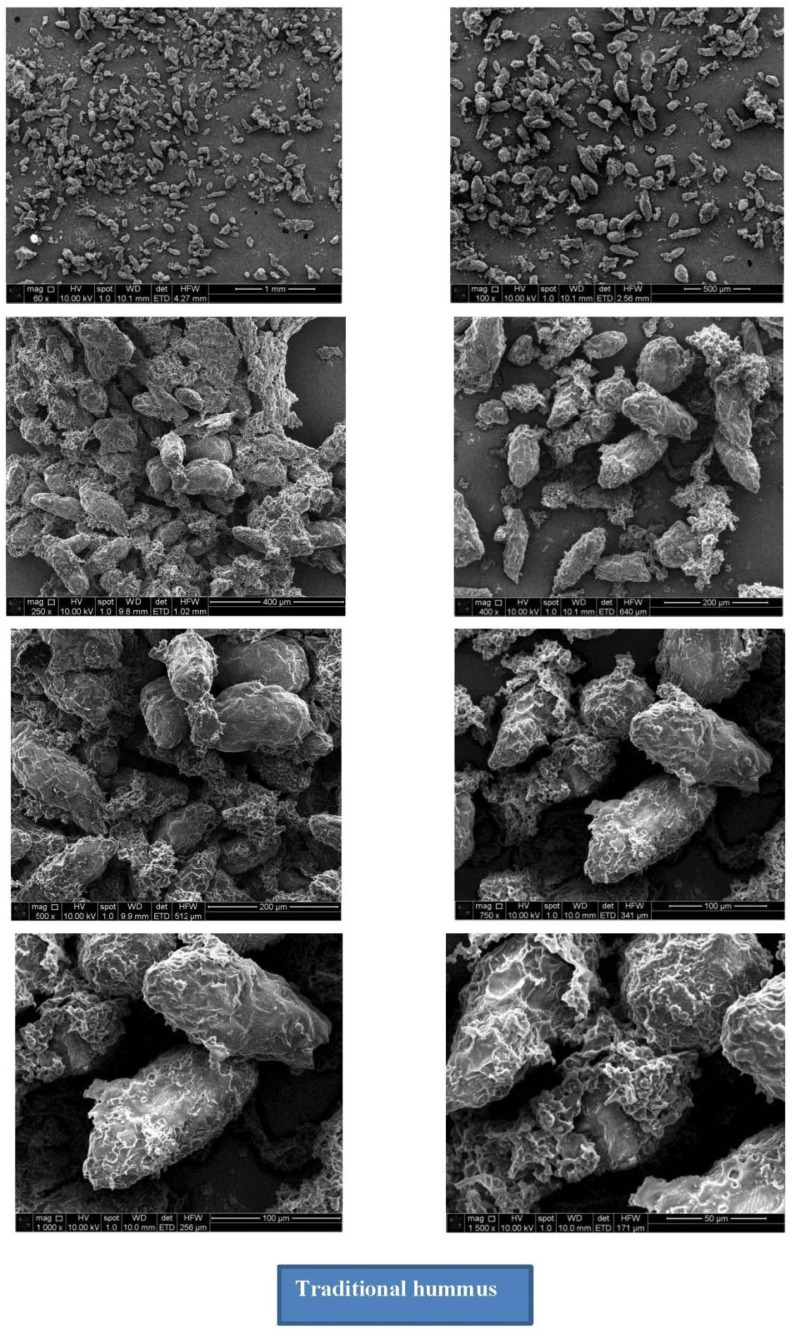
Scanning electron microscopy (SEM) of traditional hummus.

**Figure 3:**
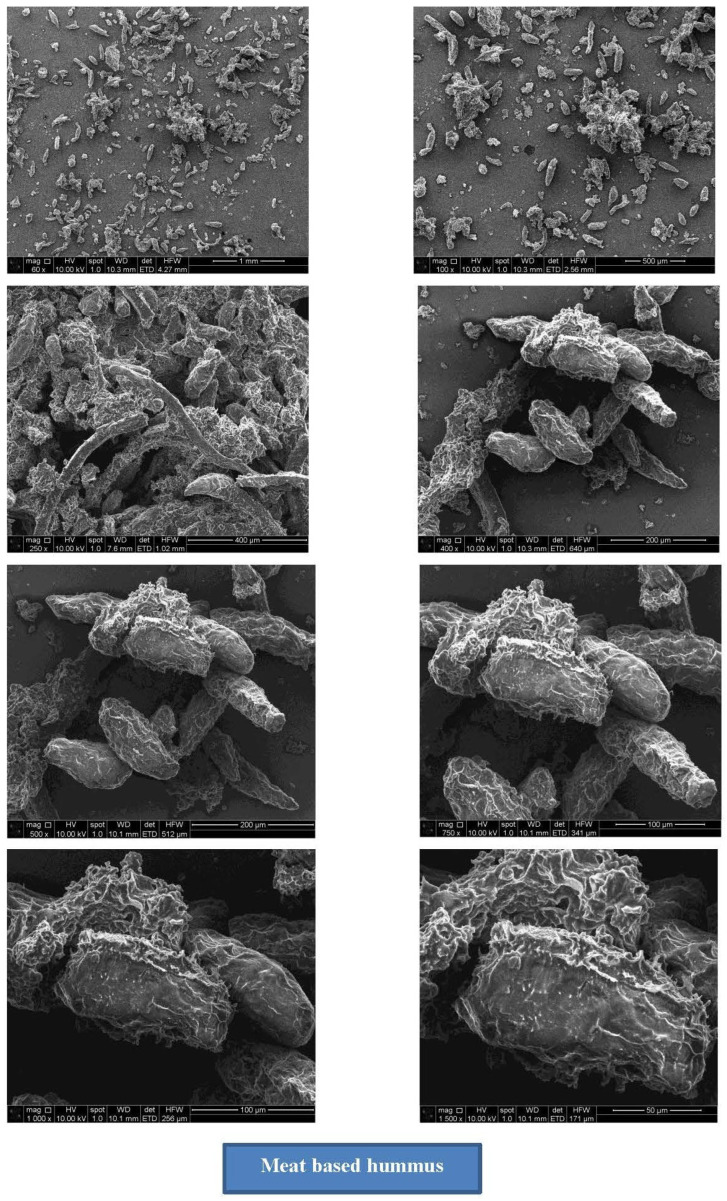
Scanning electron microscopy (SEM) of meat-based hummus.

The structural composition of traditional hummus and meat-based hummus is presented in Figure 2 and Figure 3, respectively. The microstructural differences observed under scanning electron microscopy (SEM) between traditional (plant-based chickpea hummus) and meat-based hummus highlighted the fundamental physicochemical contrasts inherent to their respective matrices. Traditional hummus displayed a porous, open network structure with irregular voids and loosely bound particles, indicating a gel-like, spreadable consistency formed primarily by plant polysaccharides, starches, and soluble fibers. This porous morphology supported high water retention from chickpea protein–polysaccharide interactions and emulsion stability [31]. Additionally, the starch granules in legumes, such as chickpeas, exhibit variable gelatinization, contributing to the bulkiness and softness of the texture [32], which aligns with the amorphous surface seen in the SEM images. Meat-based hummus exhibited a compact, fibrous, and densely structured matrix, characterized by tightly bound protein aggregates and fewer voids, which might be due to a highly structured gel network likely formed from denatured myofibrillar proteins [33] and cross-linked muscle fibers. The incorporation of 0.4% sodium acid sulfate played a key role in this structural transformation. As an acidulant, sodium acid sulfate may lower the pH, thereby enhancing protein solubility and cross-linking, which increases gel strength and water-binding capacity. Protein networks in meat emulsions resulted in more elastic, firmer textures [34], which were evident in the SEM depiction of the meat-based hummus in the present study. Moreover, the reduced porosity and more uniform distribution of lipid and protein phases in the meat variant are indicative of enhanced emulsification and phase stability, a typical feature of meat emulsions subjected to acidification and thermal processing [34].

### 3.4. Sensory Evaluation

The sensory scores of freshly prepared traditional hummus and meat-based hummus are presented in Table 5. There was no difference in color and appearance scores between traditional and meat-based hummus. However, the flavor, creaminess, grain properties, and mouth-coating scores of the meat-based hummus were higher than those of the traditional hummus. Comparatively higher sensory scores are obtained in meat-based hummus, which may be due to the umami profile and fatty acid content of lamb, resulting in a more mouth-coating and flavorful product [35,36]. In addition, the gel-forming ability of myofibrillar proteins [37] and emulsion stabilization by sodium acid sulfate in meat-based hummus might be the possible reason for the higher creaminess scores in meat-based hummus compared to traditional hummus. Future sensory studies using a larger sample size and samples collected at various storage intervals are essential for assessing the quality of meat-hummus.

## 4. Conclusions

Meat-based hummus was prepared by incorporating 50% minced lamb cooked under steam without pressure. Meat-based hummus has higher protein, fat, and bioavailable iron content, while traditional hummus has more carbohydrates and fiber. Meat-based hummus may be a suitable option for young children, athletes, and women with higher nutritional requirements, as it supports growth and muscle recovery. The microstructural analysis showed a compact, fibrous matrix in meat-based hummus and a porous structure in traditional hummus. The addition of SAS to meat-based hummus lowered the pH and resulted in a lower total plate count during storage. Although the level is minimal, the addition of mutton leads to more saturated fat. Sensory evaluation revealed higher scores for meat-based hummus in terms of flavor, creaminess, and overall acceptability. These findings suggest that meat-based hummus contains 66.6% more protein and has a lower total plate count (due to SAS) compared to traditional chickpea hummus. Therefore, developing food products that combine meat- and plant-based ingredients can provide the benefits of both, offering a nutritious option for flexitarians.

## Figures and Tables

**Table 1:** Formulation of traditional hummus and meat-based hummus.

Ingredients (%)	Traditional Hummus (%)	Meat-Based Hummus (%)
Boiled chickpea paste	70.0	35.0
Steam-cooked ground lamb meat	0.0	35.0
Tahini paste (70% toasted sesame seeds + 30% olive oil)	10.0	10.0
Water	6.0	6.0
Olive oil	7.0	7.0
Salt	1.0	1.0
Fresh garlic paste	1.0	1.0
Fresh lemon juice	5.0	4.6
Sodium acid sulfate	0.0	0.4
Total	100	100

**Table 2:** Physicochemical properties of traditional hummus and meat-based hummus.

Parameter	Traditional Hummus	Meat-Based Hummus
pH	5.65 ± 0.01	4.76 ± 0.01
L*	77.97 ± 0.31	63.80 ± 0.48
a*	10.06 ± 0.32	9.90 ± 0.05
b*	29.67 ± 0.07	22.88 ± 0.24

Mean and standard deviation are indicated. Meat-based hummus with 50% cooked minced mutton and 0.4% SAS. L* indicates lightness, with smaller values representing darker color and larger values signifying lighter color. a* indicates red to green color, with a positive value representing red color and a negative value representing green color. b* indicates blue to yellow color, with a positive value representing yellow color and a negative value representing blue color.

**Table 3:** Nutritional information for traditional hummus and meat-based hummus in one serving size of 32 g.

	Traditional Hummus	% Daily Value	Meat-Based Hummus	% Daily Value
Calorie	80		90	
Total fat	5 g	6	6 g	8
Saturated fat	0.5 g	3	1 g	5
Cholesterol	0 mg	0	10 mg	3
Sodium	130 mg	6	150 mg	7
Total carbohydrates	7 g	3	4 g	1
Dietary fiber	2 g	7	1 g	4
Protein	3 g		5 g	
Calcium	17 mg	2	14 mg	2
Iron	1 mg	6	1 mg	6
Potassium	68 mg	2	77 mg	2

Nutritional information was calculated using Genesis R&D Food Formulation Software based on the ingredients added to hummus. The % daily value was calculated on a daily diet of 2000 calories based on general nutrition advice.

**Table 4:** Nutritional information for traditional hummus and meat-based hummus in 100 kcal.

	Traditional Hummus	Meat-Based Hummus
Calorie	100.00	100.00
Total fat (g)	6.25	6.66
Saturated fat (g)	0.63	1.11
Cholesterol (mg)	0.00	11.10
Sodium (mg)	162.50	166.50
Total carbohydrates (g)	8.75	4.44
Dietary fiber (g)	2.50	1.11
Protein (g)	3.75	5.55
Calcium (mg)	21.25	15.54
Iron (mg)	1.25	1.11
Potassium g	85.00	85.47

**Table 5:** Sensory evaluation of freshly prepared traditional and meat-based hummus.

Attribute	Traditional Hummus	Meat-Based Hummus
Color and appearance	6.70 ± 0.31	6.82 ± 0.42
Flavor	6.23 ± 0.63	7.59 ± 0.34
Creaminess	6.81 ± 0.40	7.46 ± 0.32
Grain properties	5.63 ± 0.42	7.73 ± 0.37
Mouth coating	6.42 ± 0.34	7.76 ± 0.24

Mean and standard deviation are indicated. Meat-based hummus with 50% cooked minced mutton and 0.4% SAS.

## Data Availability

The original contributions presented in the study are included in the article, further inquiries can be directed to the corresponding authors.
